# Renal resistive index and aortic knob width relationship as a predictor of renal prognosis in essential hypertension

**DOI:** 10.1097/MD.0000000000012434

**Published:** 2018-10-05

**Authors:** Nurhayat Ozkan Sevencan, Aysegul Ertinmaz Ozkan

**Affiliations:** The University of Karabuk, Medical Faculty, Department of Internal Medicine, Karabuk, Turkey.

**Keywords:** aortic knob width, essential hypertension, renal prognosis, renal resistive index

## Abstract

The scientific studies that have been conducted so far highlight that renal resistive index (RI) and aortic knob width (AKW) indicate poor prognosis regarding renal and cardiovascular mortality. But the existence of a direct relationship RI and AKW is unclear. This study aims investigating the relationship between RI and the measured AKW in chest radiography of the patients with hypertensive nephropathy who do not require renal replacement therapy.

This prospective study included 268 consecutive patients with essential hypertension. Patients were divided into 2 groups as RI ≥0.7 and RI <0.7. The ROC curve, sensitivity, and specificity ratios were evaluated to determine which AKW value is the best predictive one for the RI ≥0.7.

The cutoff point of AKW was evaluated as ≥36 for the cases with RI ≥0.7: sensitivity was 71.22%; specificity was 71.32%; the positive predictive value (PPV) was 72.79; the negative predictive value (NPV) was 69.70, and the accuracy was 71.27. Area under the ROC curve ± standard error (AUC ± SE) = 0.729 ± 0.031 (*P* < .001).

AKW can provide important predictive information about the subclinical renal dysfunction in hypertensive patients with RI ≥0.7. Moreover; AKW is a predictive factor for both the diagnostic and prognostic evaluation of renal pathologies.

## 1 Introduction

Hypertension is a very common pathology in society; and an important risk factor in the development of cardiovascular diseases, renal failure, and cerebrovascular events.^[1]^ This process, which begins to manifest itself with microalbuminuria, results in severe end-organ damage over the years.^[2]^ Regarding the diagnostic and prognostic aspects, currently, renal artery doppler ultrasonography (USG) and renal resistive index (RI) measurements are in use as noninvasive methods in the evaluation of both the renal and cardiovascular pathologies that are caused by hypertension. But the inadequacy in obese and noncooperated patients and also the required experience are the disadvantages of doppler USG.^[3–5]^ Furthermore, the necessity of making an appointment for this examination and applying to the hospital for the second time constitutes another important disadvantage for the patients under the circumstances of our country.

Aortic knob width (AKW) is a measurement of radiographic structure formed by the foreshortened aortic arch and a portion of the descending aorta. Chest radiography is an inexpensive imaging method that can routinely be used to evaluate cardiovascular diseases. The close relationship between AKW and cardiovascular disease in hypertensive patients has been shown in many studies.^[6–9]^ However, it is not clear whether there is a direct relationship between AKW and RI or not.

To our knowledge, there is no study in the literature evaluating the association of AKW and RI in hypertensive nephropathic patients who do not require renal replacement therapy. AKW measurement on chest radiography is a more practical, cheaper method than doppler USG and can be easily performed by a single physician in the outpatient clinic setting. Therefore, we investigated whether AKW is associated with RI assessed by doppler USG in hypertensive nephropathic patients.

## 2 Materials and methods

### 2.1 Design and patients

A total of 268 patients aged ≥40 years with essential hypertension and applied to the Internal Medicine outpatient clinic of Karabuk University Medical Faculty Hospital between January 2017 and December 2017 were included in the study. Hypertension is defined as the measured blood pressure ≥140/90 mmHg and at least 1 anti-hypertensive drug use. Exclusion criteria were as follows:

1. Patients whose optimal doppler measurements could not be performed because of the inability of holding their breath or severe obesity.
2. Renovascular, malignant, or endocrine hypertension.^[10–12]^
3. Stenosis of renal arteries identified by doppler ultrasound.^[13]^
4. Serum creatinine >2.0 mg/dL.
5. Estimated glomerular filtration rate (eGFR) <30 mL/min/1.73 m^2^.
6. Heart failure (New York Heart Association class III or IV).
7. Congenital heart disease, systemic inflammatory disease, and Marfan syndrome.
8. Moderate to severe heart valve disease.
9. Liver cirrhosis, chronic obstructive pulmonary disease, diabetes mellitus, and presence of a neoplasm.

Moreover, we excluded the patients whose chest x-ray was not correctly centered, the patients with tracheal deviation or mediastinum shift, and any known disease in the aorta such as aortitis. All patients were informed and had given written consent forms. The study protocol fulfilled the ethical guidelines of the Declaration of Helsinki (http://www.wma.net/e/policy/b3.htm) and was approved by the local review board (Karabuk University Ethics Committee approved, date: 29.03.2017, issue: 3/1)

### 2.2 Clinical and laboratory evaluation

**Figure 1:**
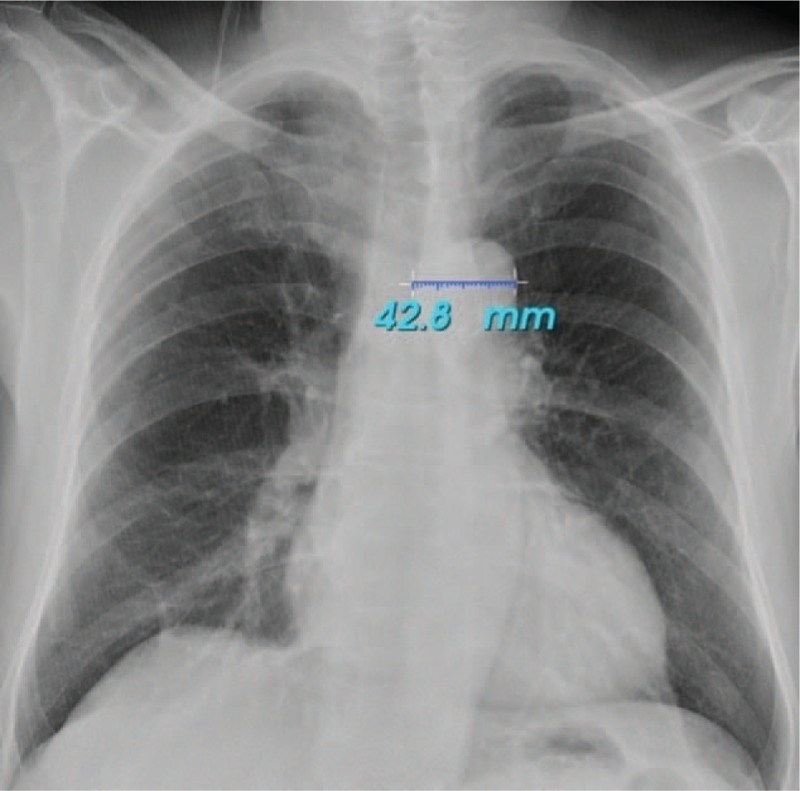
AKW measurement method in chest radiography.

Clinical history was obtained from all patients, and physical examinations were performed. A nurse measured body weights and heights. Blood pressure was measured in the sitting position after 5 minutes of rest via a mercury sphygmomanometer according to the standard recommendations. The blood pressure was measured and recorded by a physician with a certain electronic oscillometric device (Perfect-Aneroid sphygmomanometer, ERKA, Germany).^[14]^ Routine biochemical parameter determination was performed with the standard techniques by using an autoanalyzer. eGFR was calculated by using the Chronic Kidney Disease Epidemiology Collaboration Equation.^[15]^ A radiologist performed all the doppler examinations with a color doppler USG. Peak-systolic velocity (PSH) and end-diastolic velocity (EDH) were measured from the interlobular branches of the renal arteries with the appropriate angle, and the mean RI was calculated (RI = [PSH−EDH] / PSH). For the assessment of AKW, posteroanterior chest x-ray was performed on all the patients. A single physician who did not know the aims of the study and blinded to the patient's data examined the chest radiography. The AKW was measured along the horizontal line from the point of the lateral edge of the trachea to the left lateral wall of the aortic knob (Fig. 1).

### 2.3 Statistical analysis

NCSS (Number Cruncher Statistical System) v.2007 (Kaysville, UT) program was used for the statistical analysis. As well as the descriptive statistical methods (mean, standard deviation, median, frequency, ratio, minimum, maximum), student *t* test was used for the quantitative comparisons of the 2 groups with normal data distribution, and Mann-Whitney *U* test was used for the comparison of 2 groups with abnormal data distribution. Pearson *χ*^2^ test was used for the comparison of the qualitative data. Diagnostic screening tests (sensitivity, specificity, PPV, NPV) and ROC curve analysis were used to determine the cutoff value for the parameters. Pearson correlation test was performed for the correlation matrix. The significance level was *P* < .05.

The area under the curve (AUC) is not dependent on a specific operating point but describes the whole area under the ROC curve. The AUC is an effective and combined measure of the sensitivity and specificity, which also describes the inherent validity of diagnostic tests. The maximum AUC = 1 means that the diagnostic test is adequate in the differentiation of the disease presence and absence.^[16]^

We have chosen a cutoff value of 0.7 for renal RI because it has been demonstrated in previous studies that the subjects with a renal RI above this value had an increased prevalence of hypertensive end-organ damage and a faster progression of renal diseases.^[17–20]^

## 3 Results

The patients with RI ≥0.7 were significantly older than the patients with RI <0.7 (*P* = .001). There was no statistically significant difference regarding the other demographic characteristics of the patients. The AKW measurements of subjects with RI ≥0.7 were significantly higher than those with RI <0.7 (*P* = .001). eGFR measurements of RI ≥0.7 were significantly lower than those of with RI <0.7 (*P* = .001). There was no statistically significant difference between the groups of RI regarding the biochemical tests. The demographic characteristics and biochemical test results of the patients according to the RI groups are shown in Table 1.

**Table 1:**
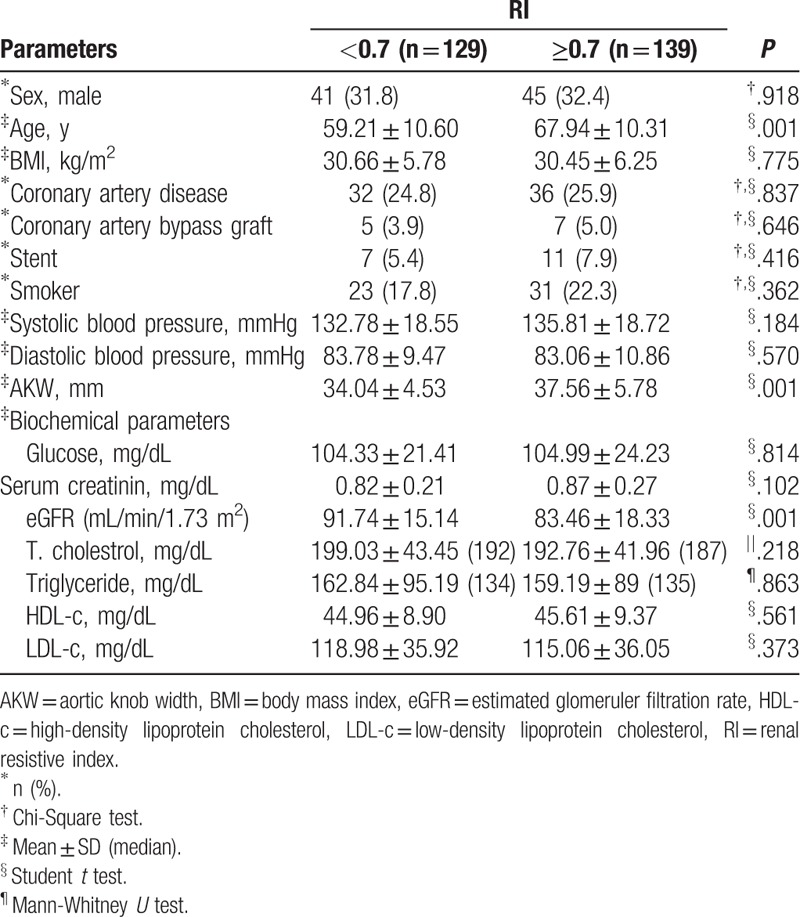
Demographic characteristics and biochemical parameters of patients according to RI groups.

**Figure 2:**
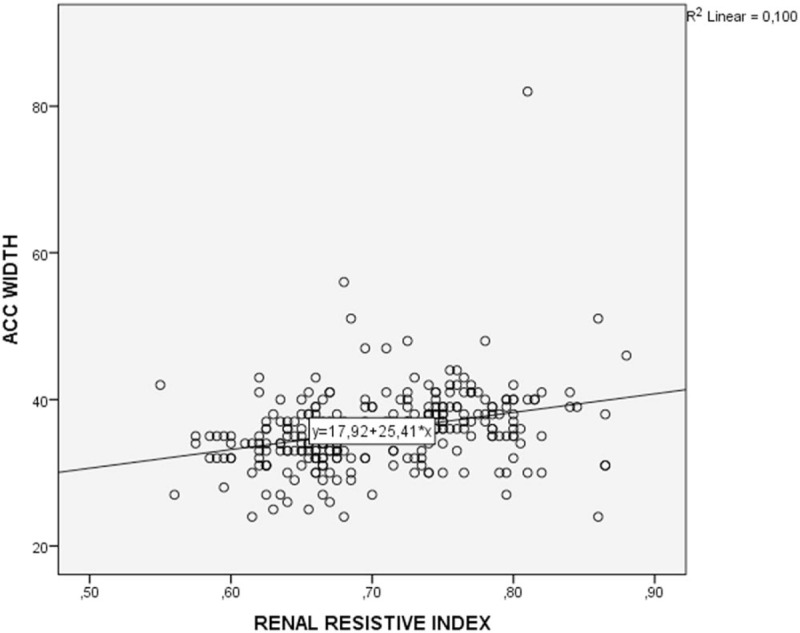
Correlation between AKW and RI.

**Figure 3:**
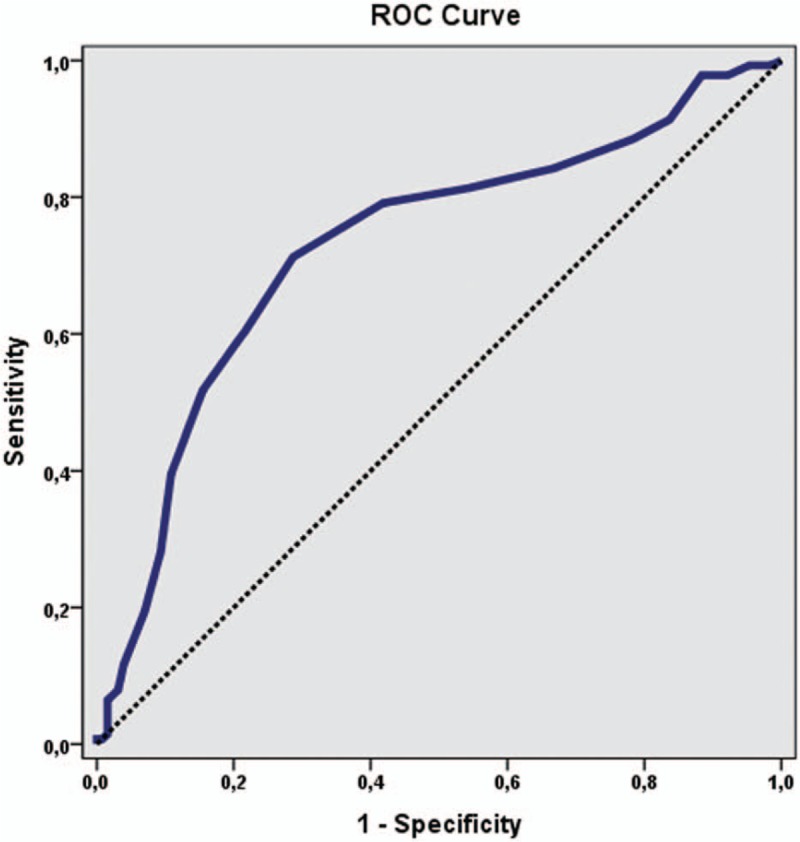
ROC curve for AKW level in patients with RI ≥0.7.

The cutoff point of AKW was evaluated as 36 and above for the cases with RI ≥0.7: sensitivity was 71.22%; specificity was 71.32%; the positive predictive value was 72.79; the negative predictive value was 69.70, and the accuracy was 71.27. There was a statistically significant moderate strength, and a positive correlation between RI and AKW (*r* = 316, *P* < .001) (Fig. 2). In the obtained ROC curve, the underlying area was found to be 72.9%, and the standard error was 3.1%. Diagnostic screening tests and ROC curve results for AKW are shown in Table 2 and Figure 3.

**Table 2:**
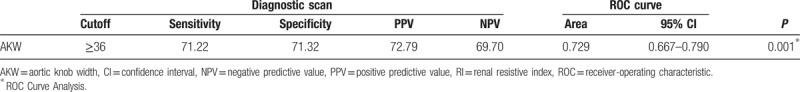
Diagnostic screening tests and ROC curve results for AKW in patients with RI ≥0.7.

## 4 Discussion

We investigated whether there was a relationship between RI and AKW in patients with hypertensive nephropathy and also who did not require renal replacement therapy. To our knowledge, there is no study evaluating the association of AKW and RI directly in the literature. Therefore, our research is the first one to study this relationship. This study showed that in the group with RI ≥0.7, the eGFR was significantly lower and the AKW was significantly increased. The ROC curve analysis revealed that a 36 mm of AKW produced a cutoff value of RI ≥0.7 with 71.22% sensitivity and 71.32% specificity.

Increased RI as measured by doppler USG has been shown to be in correlation with the severity of renal failure in hypertensive patients. In a study conducted by Doi et al, the prognostic role of RI in cardiovascular and renal outcome was investigated. A total of 426 essential hypertensive patients without cardiovascular disease were included in the study. The combination of high RI and low eGFR has been found to be a strong predictor of cardiovascular and renal poor outcomes in patients with essential hypertension.^[3]^ In another study by Doi et al, RI was found to be associated with higher levels of albuminuria and left ventricular hypertrophy in patients with essential hypertension. These results indicate that RI may be a predictor of subclinical end-organ damage.^[18]^

In a study by Toledo et al, 1962, chronic renal disease patients without renal artery stenosis were followed for 2.2 years and the factors associated with higher RI were evaluated. Those with RI ≥0.70 were associated with higher mortality rates at a significant level, and a 0.05-unit increase in RI was significantly in correlation with high mortality risk.^[19]^ In the study of Tanimura et al, which consisted of 175 hypertensive patients, a statistically significant correlation was found not only between eGFR and anemia but also between RI and anemia. A value of RI >0.7 was found to be a predictive factor for the presence and severity of anemia. A 0.05-unit increase in RI was significantly in correlation with high mortality risk.^[20]^ In the context of these previous studies, we considered the cutoff point of RI value as 0.7 between the groups. In our study, the group with RI ≥0.7 was associated with older age and lower eGFR.

The relationship between AKW and atherosclerosis was first introduced in 1979. Fiore et al investigated AKW on 691 chest radiograms and reported that AKW was increased in atherosclerotic, hypertensive, and advanced aged patients.^[21]^ In the study of Yun et al, 178 patients AKW and aortic arch calcification measurements were examined, and these parameters were in correlation with the coronary artery disease severity.^[22]^ Rayner et al found that the cardiothoracic ratio and AKW which of both can be measured on the chest radiography, were significant predictors of target organ damage.^[23]^ In the study of Gürbak et al,^[9]^ the relationship between AKW and left ventricular hypertrophy was evaluated in hypertensive patients, and AKW was found to be an essential indicator of subclinical left ventricular hypertrophy. ROC curve analysis demonstrated that aortic knob of 37 mm constitutes the cutoff value for the presence of subclinical LV dysfunction with 85.9% sensitivity and 86.4% specificity. In the study of Erkan et al,^[24]^ it was found that there was a strong correlation between AKW and carotid intima-media thickness, and AKW was found to be an essential predictor of subclinical atherosclerosis.

Afsar et al compared AKW values of hemodialysis patients with the healthy control group. Age, high pre-dialysis systolic blood pressure, male sex, being a nonsmoker, increased total cholesterol, and increased parathormone levels were independently associated with increased AKW. In this study, AKW was found to be 35.0 ± 5.8 mm for hemodialysis patients and 26.6 ± 4.3 mm for the control group.^[25]^

In our study, the cutoff value of AKW was ≥36 in the group with RI ≥0.7. Furthermore, the eGFR was statistically significantly lower in RI ≥0.7. These findings indicate that AKW is a significant predictor of high RI and low eGFR in patients who do not require renal replacement therapy as in dialysis patients.

## 5 Conclusion

In this study, we have observed that AKW and RI have a close relationship. Therefore, AKW may provide crucial predictive information on subclinical kidney disease in hypertensive patients. However, further studies with larger sample sizes and different populations are required to assess the applicability of this model.

## Author contributions

**Data curation:** Aysegul Ertinmaz Ozkan.

**Investigation:** Nurhayat Ozkan Sevencan.

**Methodology:** Nurhayat Ozkan Sevencan.

**Project administration:** Nurhayat Ozkan Sevencan.

**Resources:** Aysegul Ertinmaz Ozkan.

**Writing – original draft:** Nurhayat Ozkan Sevencan.

**Writing – review & editing:** Nurhayat Ozkan Sevencan.

Nurhayat Ozkan Sevencan orcid: 0000-0001-9013-3517.
